# Supplementary material for: Salivary microbiota reflecting changes in subgingival microbiota
Source: Microbiol Spectr. 2024 Oct 4;12(11):e01030-24. doi: 10.1128/spectrum.01030-24 (PMC11537074; doi:10.1128/spectrum.01030-24)
Supplement: Supplement 1 — Comparison of ACE, Chao1, Jackknife, and the number of identified species. [file spectrum.01030-24-s0001.pdf]

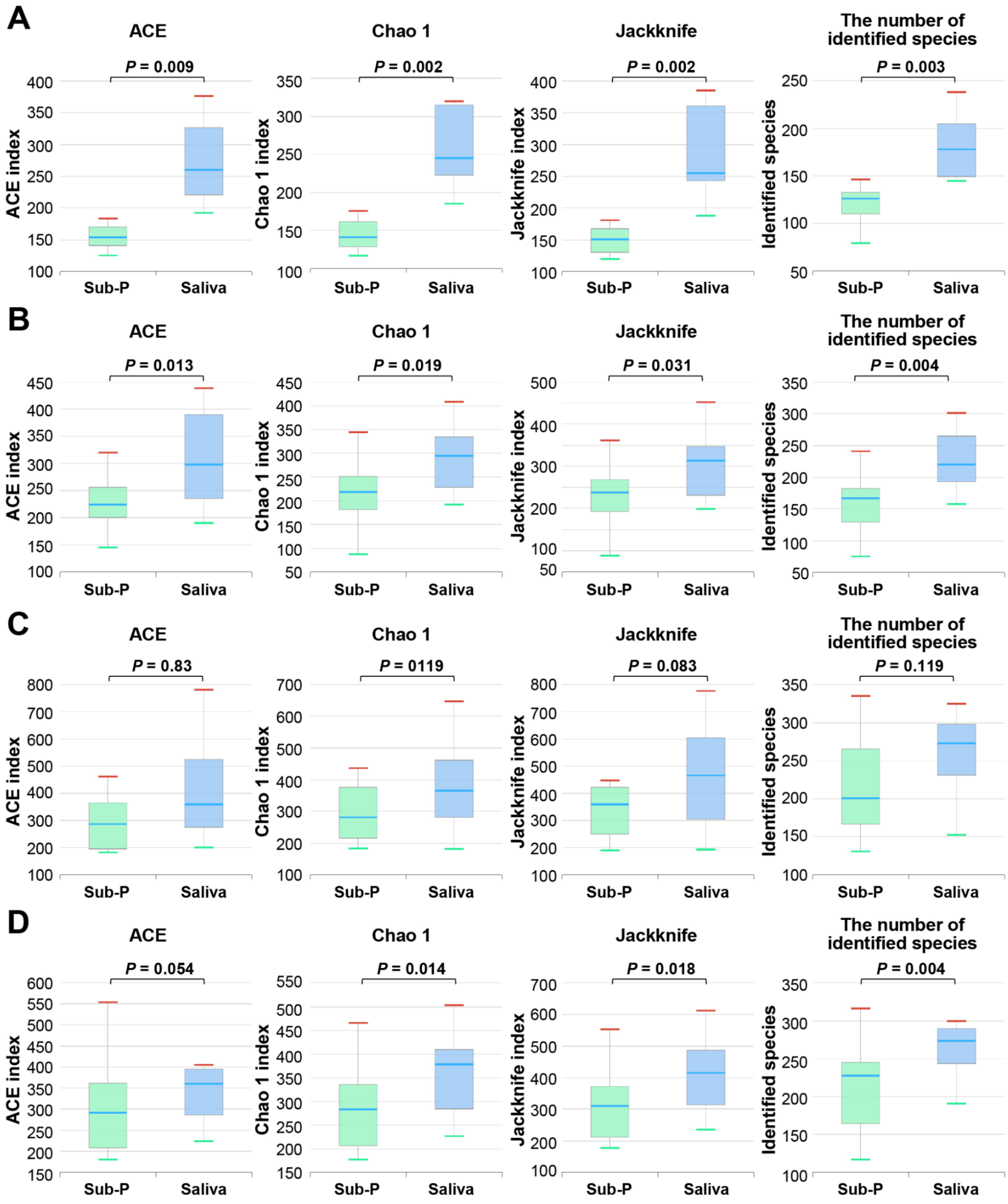

Supplement 1. Comparison of ACE, Chao1, Jackknife, and the number of identified species between subgingival plaque and saliva samples in healthy (n=7) subjects (A), those with gingivitis (n=14) (B), moderate periodontitis (n=12) (C), or severe periodontitis (n=18) (D). Each value is presented as a box plot. Top, middle, and bottom lines of the boxes represent the 25th, 50th (median), and 75th percentiles, respectively. The significance of differences between two groups was evaluated using the Wilcoxon rank-sum test, and $p < 0.05$ was considered to indicate a statistically significant difference. Sub-P, subgingival plaque.
