# Supplementary material for: Salivary microbiota reflecting changes in subgingival microbiota
Source: Microbiol Spectr. 2024 Oct 4;12(11):e01030-24. doi: 10.1128/spectrum.01030-24 (PMC11537074; doi:10.1128/spectrum.01030-24)
Supplement: Supplement 2 — B.PCoA plot illustrating beta diversity distance matrices of the Jensen-Shannon distance. [file spectrum.01030-24-s0002.pdf]

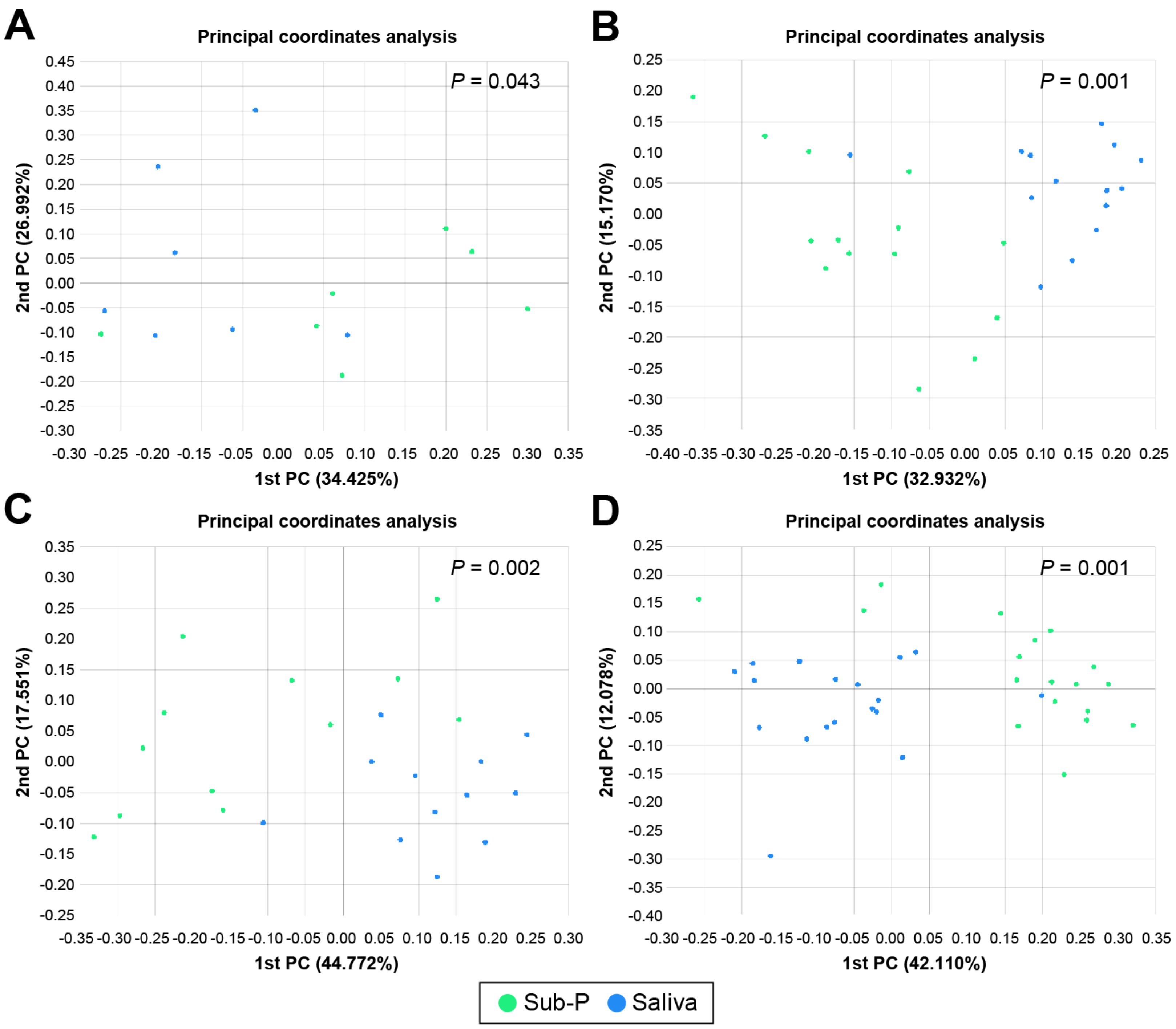

Supplement 2. B. PCoA plot illustrating beta diversity distance matrices of the Jensen-Shannon distance comparing the sample distribution for healthy subjects (n=7) (A), those with gingivitis (n=14) (B), moderate periodontitis (n=12) (C), or severe periodontitis (n=18) (D) between subgingival plaque and saliva samples. Green dots represent subgingival plaque samples, and blue dots represent saliva samples. Permutational multivariate analysis of variance (PERMANOVA) results demonstrated beta set-significance between the subgingival plaque and saliva groups. Sub-P, subgingival plaque.
