# Supplementary material for: Salivary microbiota reflecting changes in subgingival microbiota
Source: Microbiol Spectr. 2024 Oct 4;12(11):e01030-24. doi: 10.1128/spectrum.01030-24 (PMC11537074; doi:10.1128/spectrum.01030-24)
Supplement: Supplement 3 — Relative abundance of the 20 species. [file spectrum.01030-24-s0003.pdf]

Taxon name	Sample type	Health	Gingivitis	Moderate periodontitis	Severe periodontitis
Porphyromonas gingivalis	Sub-P	0.05±0.05	1.35±1.25	1.76±0.89	10.70±2.92
	Saliva	0.13±0.07	0.44±0.31	1.73±0.97	6.88±2.01
Fusobacterium nucleatum group	Sub-P	6.70±2.00	8.98±1.86	10.14±1.82	15.32±2.42
	Saliva	1.13±0.45	1.77±0.52	2.16±0.51	5.24±1.04
Tannerella forsythia	Sub-P	0.26±0.22	0.43±0.28	1.50±0.60	3.60±1.00
	Saliva	0.12±0.05	0.12±0.05	0.64±0.30	1.19±0.33
Porphyromonas endodontalis	Sub-P	0.11±0.09	1.48±0.87	2.71±1.22	3.54±1.27
	Saliva	0.06±0.02	0.37±0.11	0.89±0.33	1.58±0.35
KE332528_s	Sub-P	0.00±0.00	0.13±0.12	0.40±0.22	2.69±1.11
	Saliva	0.00±0.00	0.06±0.04	0.17±0.10	0.79±0.26
Prevotella intermedia	Sub-P	0.20±0.13	1.11±0.53	2.51±1.41	1.98±0.65
	Saliva	0.08±0.07	0.17±0.09	0.32±0.13	0.78±0.18
Treponema_uc	Sub-P	0.02±0.02	0.29±0.19	0.82±0.29	1.93±0.74
	Saliva	0.03±0.03	0.09±0.06	0.31±0.12	0.95±0.38
ADCM_s	Sub-P	0.51±0.51	0.51±0.32	1.71±0.60	1.93±1.10
	Saliva	0.11±0.10	0.29±0.12	0.19±0.07	0.37±0.22
Treponema medium group	Sub-P	0.03±0.02	0.79±0.29	1.08±0.22	1.66±0.64
	Saliva	0.05±0.03	0.19±0.07	0.34±0.17	0.59±0.16
Campylobacter showae group	Sub-P	0.06±0.02	0.72±0.33	2.65±0.99	1.19±0.37
	Saliva	0.15±0.06	0.32±0.13	0.70±0.23	0.84±0.24
GU430992_s	Sub-P	0.02±0.02	0.19±0.13	0.45±0.18	1.05±0.28
	Saliva	0.01±0.00	0.05±0.02	0.15±0.06	0.29±0.09
Mycoplasma faucium	Sub-P	0.00±0.00	0.24±0.18	0.47±0.20	1.00±0.28
	Saliva	0.00±0.00	0.16±0.08	0.32±0.20	1.07±0.32
Filifactor alocis	Sub-P	0.01±0.01	0.20±0.15	0.40±0.19	0.99±0.22
	Saliva	0.01±0.01	0.04±0.02	0.13±0.07	0.41±0.12
Haemophilus parainfluenzae group	Sub-P	8.65±4.25	6.19±2.25	1.98±0.66	1.57±1.02
	Saliva	7.74±3.02	8.49±1.19	4.27±0.90	4.91±1.24
Rothia dentocariosa	Sub-P	8.22±3.75	2.11±1.03	1.11±0.41	1.50±0.62
	Saliva	2.53±1.16	0.97±0.35	0.35±0.18	0.89±0.42
Lautropia mirabilis	Sub-P	4.30±3.06	4.14±2.00	2.97±1.50	1.19±0.56
	Saliva	3.27±2.08	1.24±0.39	2.06±0.64	0.63±0.28
Neisseria subflava	Sub-P	5.27±3.68	0.88±0.48	1.95±0.90	0.81±0.61
	Saliva	9.96±3.28	5.86±1.73	5.87±1.58	3.90±1.03
KV831974_s group	Sub-P	4.38±2.34	0.82±0.56	0.89±0.37	0.12±0.08
	Saliva	6.95±2.96	2.45±0.40	3.07±0.82	1.90±0.77
Streptococcus sanguinis group	Sub-P	2.27±0.75	3.48±1.49	2.16±0.72	1.07±0.73
	Saliva	4.53±2.84	3.25±1.66	1.79±0.57	1.17±0.31
Streptococcus sinensis group	Sub-P	1.89±1.48	0.20±0.12	0.73±0.39	0.08±0.04
	Saliva	4.84±1.63	2.25±0.44	1.67±0.37	2.52±1.61

Supplement 3. Relative abundance of the 20 species present at levels greater than 1% of the bacteria that differed among the four groups by disease severity. Relative abundance according to disease severity in subgingival plaque (Sub-P) and saliva samples was expressed as mean ± standard deviation.
