# Supplementary material for: Salivary microbiota reflecting changes in subgingival microbiota
Source: Microbiol Spectr. 2024 Oct 4;12(11):e01030-24. doi: 10.1128/spectrum.01030-24 (PMC11537074; doi:10.1128/spectrum.01030-24)
Supplement: Supplement 4 — A heat map distribution of the 20 species. [file spectrum.01030-24-s0004.pdf]

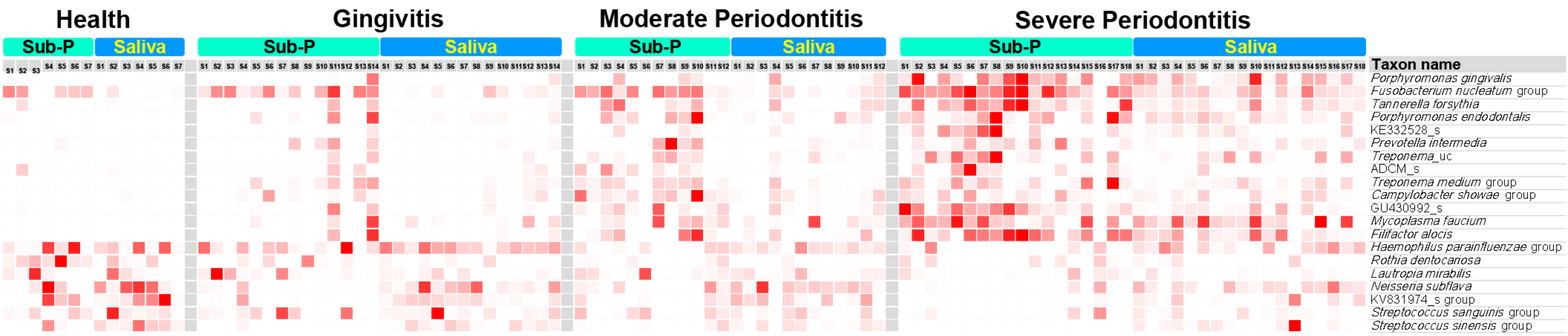

Supplement 4. A heat map distribution of the 20 species present at levels greater than 1% of the bacteria that differed among the four groups by disease severity. Relative abundance of each species in subgingival plaque (Sub-P) and saliva samples is indicated by the red gradient in each grid (light = low abundance; dark = high abundance). Grids located on the left and right sides of each group (health, gingivitis, moderate periodontitis, and severe periodontitis groups) indicate the distribution in the subgingival plaque and saliva samples, respectively. Sub-P, subgingival plaque.
