# Supplementary material for: Salivary microbiota reflecting changes in subgingival microbiota
Source: Microbiol Spectr. 2024 Oct 4;12(11):e01030-24. doi: 10.1128/spectrum.01030-24 (PMC11537074; doi:10.1128/spectrum.01030-24)
Supplement: Supplement 5 — Pre- and post-treatment levels of taxa showing significant difference for all 14 periodontitis patients in subgingival plaque. [file spectrum.01030-24-s0005.pdf]

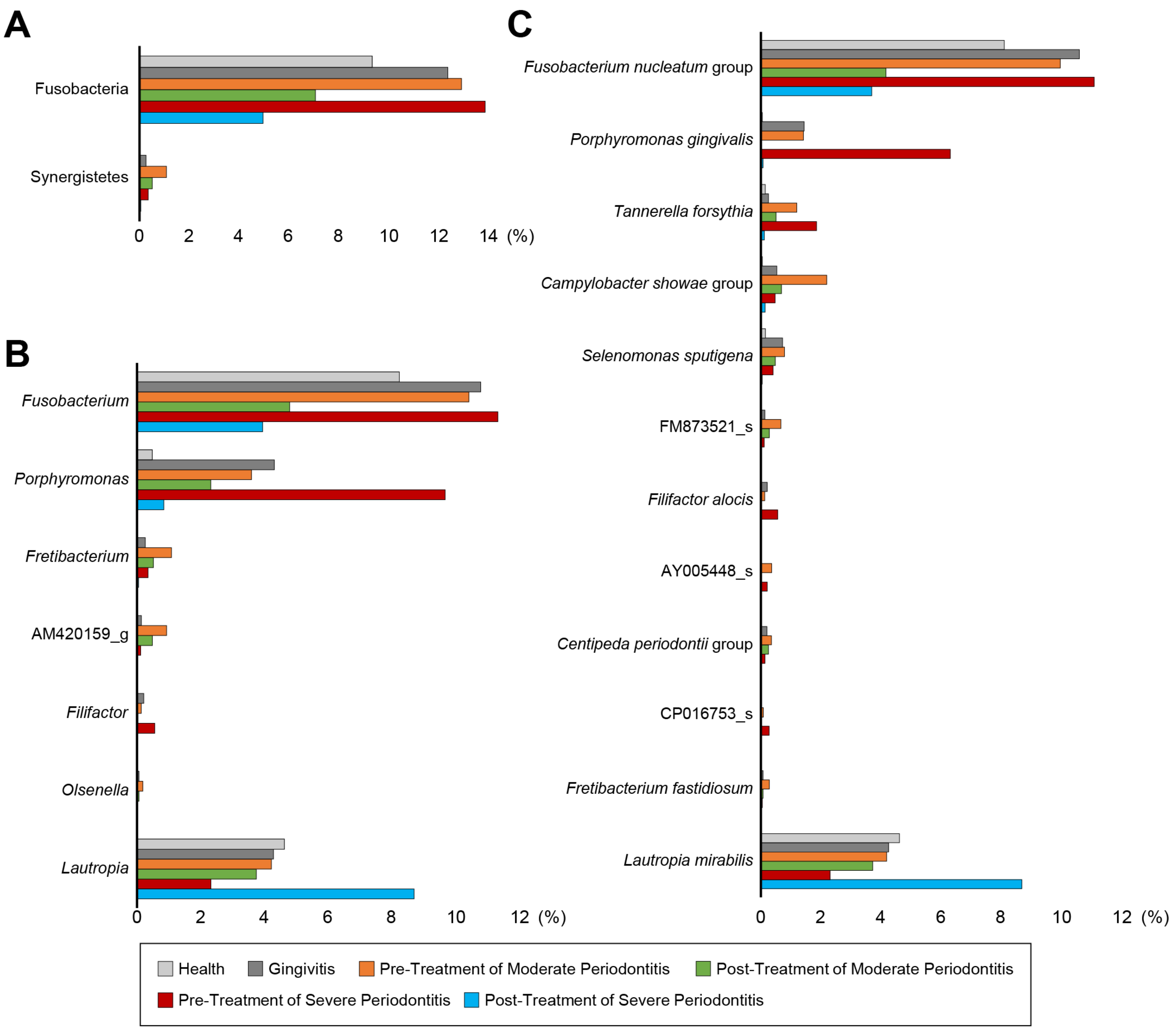

Supplement 5. Pre- and post-treatment levels of taxa showing statistically significant difference pre- and post-treatment for all 14 periodontitis patients in subgingival plaque samples of moderate (n=7) or severe periodontitis (n=7) subjects. Relative abundances are displayed along with those in the H and G groups. A. Relative abundance of phyla in pre- and post-treatment samples from moderate and severe periodontitis patients. B. Relative abundance of genera pre- and post-treatment samples from subjects with moderate or severe periodontitis. The six genera listed at the top were dominant pre-treatment, while the one genus listed at the bottom was dominant after treatment among genera > 0.01% in the subgingival plaque samples. C. Relative abundance of species in pre- and post-treatment samples from subjects with moderate or severe periodontitis. The 11 species listed at the top were dominant in the pre-treatment samples, while the one species listed at the bottom was dominant after treatment among species > 0.01% in the subgingival plaque samples. $p < 0.05$ by Kruskal–Wallis H test comparing pre- and post-treatment samples.
