# Supplementary material for: Salivary microbiota reflecting changes in subgingival microbiota
Source: Microbiol Spectr. 2024 Oct 4;12(11):e01030-24. doi: 10.1128/spectrum.01030-24 (PMC11537074; doi:10.1128/spectrum.01030-24)
Supplement: Supplement 6 — Pre- and post-treatment levels of taxa showing saliva samples from 14 periodontitis patients. [file spectrum.01030-24-s0006.pdf]

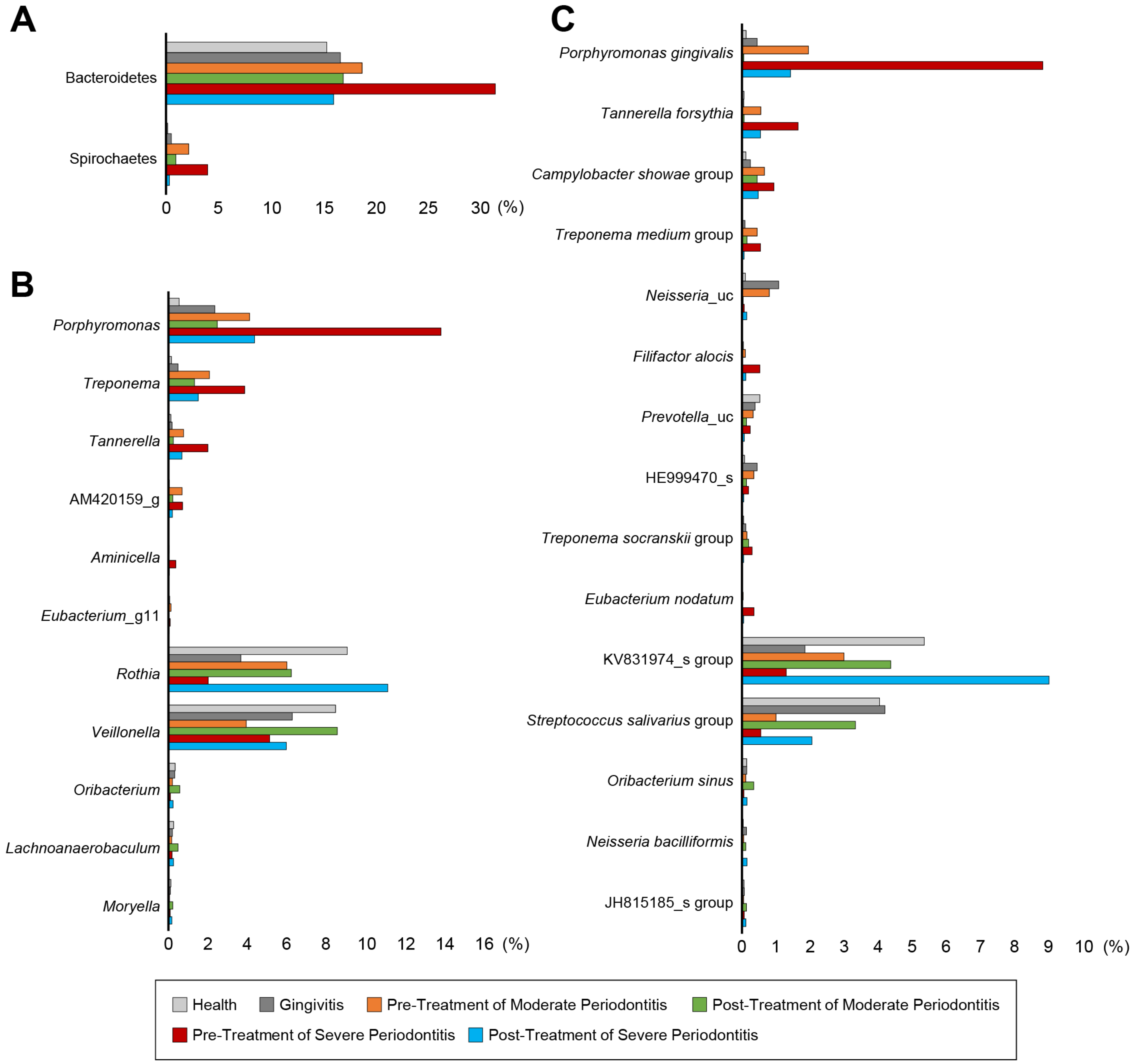

Supplement 6. Pre- and post-treatment levels of taxa showing statistically significant difference pre- and post-treatment in saliva samples from 14 periodontitis patients with moderate (n=7) or severe periodontitis (n=7). Their relative abundances are displayed along with those in the H and G groups. A. Relative abundance of phyla in pre- and post-treatment samples from subjects with moderate or severe periodontitis. B. Relative abundance of genera in pre- and post-treatment samples from subjects with moderate or severe periodontitis. The six genera listed at the top were dominant pre-treatment, while the five genera listed at the bottom were dominant after treatment among genera > 0.01% in the salvia samples. C. Relative abundance of species in pre- and post-treatment samples from subjects with moderate and severe periodontitis. The 11 species listed at the top were dominant pre-treatment, while the five species listed at the bottom were dominant after treatment among species > 0.01% in the salvia samples. $p < 0.05$ by Kruskal–Wallis H test comparing pre- and post-treatment samples.
