# Supplementary material for: Salivary microbiota reflecting changes in subgingival microbiota
Source: Microbiol Spectr. 2024 Oct 4;12(11):e01030-24. doi: 10.1128/spectrum.01030-24 (PMC11537074; doi:10.1128/spectrum.01030-24)
Supplement: Supplement 7 — Correlation in rate of reduction of phyla, genera, and species in subgingival plaque and saliva samples. [file spectrum.01030-24-s0007.pdf]

		Spearman R	*P*-value
**Phylum**	Bacteroidetes*	0.604	0.022
	Fusobacteria	0.240	0.409
	Spirochaetes	0.000	1.000
	Synergistetes	-0.006	0.986
**Genus**	AM420159_g	0.522	0.082
	Aminicella	0.277	0.547
	*Eubacterium*_g11	-0.005	0.988
	Filifactor	0.532	0.114
	Fretibacterium	-0.218	0.519
	Fusobacterium	0.204	0.483
	Lachnoanaerobaculum	0.036	0.915
	Lautropia	0.217	0.499
	Moryella	0.200	0.578
	Olsenella	-0.264	0.461
	*Oribacterium**	0.669	0.035
	*Porphyromonas**	0.745	0.002
	Rothia	0.138	0.637
	Tannerella	0.231	0.427
	Treponema	0.143	0.642
	Veillonella	0.415	0.140
**Species**	AY005448_s	0.135	0.773
	Campylobacter showae group*	0.752	0.020
	Centipeda periodontii group	0.402	0.154
	CP016753_s	0.225	0.592
	Eubacterium brachy	-0.005	0.988
	Eubacterium nodatum	0.277	0.547
	Filifactor alocis	0.515	0.127
	FM873521_s	0.532	0.114
	*Fretibacterium fastidiosum**	0.772	0.009
	Fusobacterium nucleatum group	0.310	0.281
	HE999470_s	0.408	0.166
	JH815185_s group	0.191	0.574
	KV831974_s group	0.495	0.086
	Lautropia mirabilis	0.217	0.499
	*Neisseria*_uc	0.302	0.430
	Oribacterium sinus	0.353	0.317
	Porphyromonas gingivalis	0.366	0.269
	*Prevotella*_uc*	0.862	0.001
	Selenomonas sputigena	0.462	0.112
	Streptococcus salivarius group	0.413	0.161
	Tannerella forsythia	0.109	0.737
	Treponema medium group	0.283	0.348
	Treponema socranskii group	0.453	0.139
	P. g + T. f + F.a*	0.571	0.033

Supplement 7. Correlation in rate of reduction of phyla, genera, and species in subgingival plaque and saliva samples. Spearman coefficient (r) and P value are indicated. $^{*}p < 0.05$ by Spearman correlation between pre- and post-treatment periodontitis samples. P.g+T.f+F.a indicates the sum of the reduction ratios of *P. gingivalis* (P.g), *T. forsythia* (T.f), and *F. alocis* (F.a).
