# Supplementary material for: Salivary microbiota reflecting changes in subgingival microbiota
Source: Microbiol Spectr. 2024 Oct 4;12(11):e01030-24. doi: 10.1128/spectrum.01030-24 (PMC11537074; doi:10.1128/spectrum.01030-24)
Supplement: Supplement 8 — Panoramic radiographs of patient. [file spectrum.01030-24-s0008.pdf]

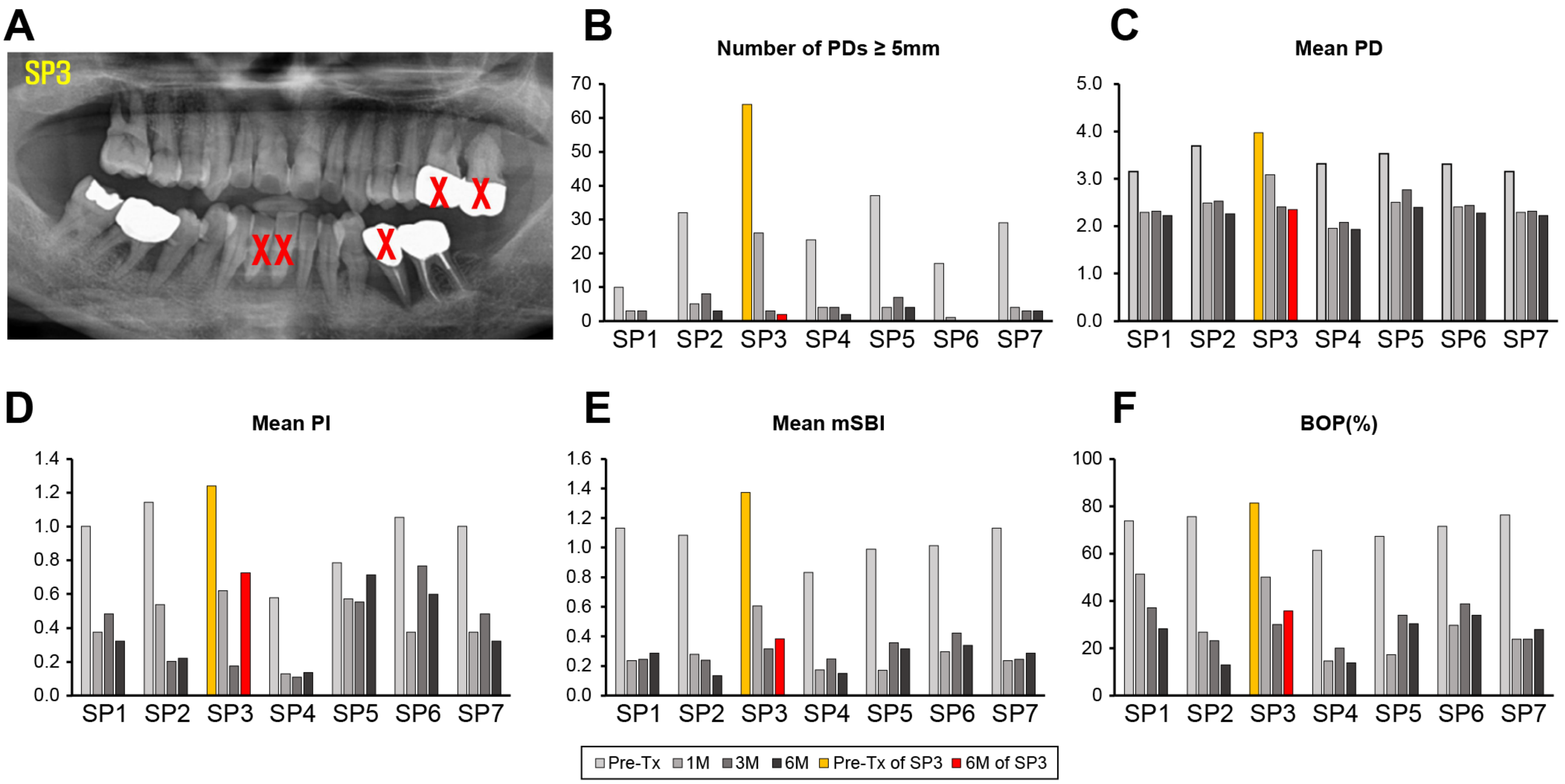


**Supplement 8.** Panoramic radiographs of patient SP3 (A) and clinical characteristics of seven patients with severe periodontitis following periodontal treatment (B~F) A. panoramic radiographs of patient SP3 baseline. Among a total of 25 teeth at baseline, 5 teeth marked with an X were extracted during the treatment. **B**. Number of sites with PD ≥ 5 mm pre-treatment (Pre-Tx) and 1 month (1M), 3 months (3M), and 6 months (6M) after treatment. **C**. Mean PD pre-treatment and at 1 month, 3 months, and 6 months after treatment. **D**. Mean PI pre-treatment and at 1 month, 3 months, and 6 months after treatment. **E**. Mean mSBI pre-treatment and at 1 month, 3 months, and 6 months after treatment. **F**. Mean BOP (%)pre-treatment and at 1 month, 3 months, and 6 months after treatment. In B~F, clinical parameters before and 6 months after treatment of subject SP3 are displayed in orange and red, respectively. PD, probing depth; PI, plaque index; mSBI, modified sulcus bleeding index; BOP, bleeding on probing
